# Better communication between rheumatologists and radiologists is key to improving MRI requesting and reporting data in axial spondyloarthritis: audit data from a single specialist centre

**DOI:** 10.1093/rap/rkag053

**Published:** 2026-04-25

**Authors:** Jake Weddell, Henry de Boer, Joshua Wong, Bill Pass, Philip Robinson, Andrew Barr, Claire Vandevelde, Jane E Freeston, Dennis McGonagle, Helena Marzo-Ortega

**Affiliations:** NIHR Leeds Biomedical Research Centre and Department of Rheumatology, Chapel Allerton Hospital, The Leeds Teaching Hospitals NHS Trust, Leeds, UK; Leeds Institute of Rheumatic and Musculoskeletal Medicine, University of Leeds, Leeds, UK; Department of Musculoskeletal Radiology, Chapel Allerton Hospital, The Leeds Teaching Hospitals Trust, Leeds, UK; Department of Musculoskeletal Radiology, Chapel Allerton Hospital, The Leeds Teaching Hospitals Trust, Leeds, UK; NIHR Leeds Biomedical Research Centre and Department of Rheumatology, Chapel Allerton Hospital, The Leeds Teaching Hospitals NHS Trust, Leeds, UK; Department of Musculoskeletal Radiology, Chapel Allerton Hospital, The Leeds Teaching Hospitals Trust, Leeds, UK; NIHR Leeds Biomedical Research Centre and Department of Rheumatology, Chapel Allerton Hospital, The Leeds Teaching Hospitals NHS Trust, Leeds, UK; Department of Musculoskeletal Radiology, Chapel Allerton Hospital, The Leeds Teaching Hospitals Trust, Leeds, UK; NIHR Leeds Biomedical Research Centre and Department of Rheumatology, Chapel Allerton Hospital, The Leeds Teaching Hospitals NHS Trust, Leeds, UK; Leeds Institute of Rheumatic and Musculoskeletal Medicine, University of Leeds, Leeds, UK; NIHR Leeds Biomedical Research Centre and Department of Rheumatology, Chapel Allerton Hospital, The Leeds Teaching Hospitals NHS Trust, Leeds, UK; Leeds Institute of Rheumatic and Musculoskeletal Medicine, University of Leeds, Leeds, UK; NIHR Leeds Biomedical Research Centre and Department of Rheumatology, Chapel Allerton Hospital, The Leeds Teaching Hospitals NHS Trust, Leeds, UK; Leeds Institute of Rheumatic and Musculoskeletal Medicine, University of Leeds, Leeds, UK; NIHR Leeds Biomedical Research Centre and Department of Rheumatology, Chapel Allerton Hospital, The Leeds Teaching Hospitals NHS Trust, Leeds, UK; Leeds Institute of Rheumatic and Musculoskeletal Medicine, University of Leeds, Leeds, UK; NIHR Leeds Biomedical Research Centre and Department of Rheumatology, Chapel Allerton Hospital, The Leeds Teaching Hospitals NHS Trust, Leeds, UK; Leeds Institute of Rheumatic and Musculoskeletal Medicine, University of Leeds, Leeds, UK

**Keywords:** Axial spondyloarthritis, Magnetic Resonance Imaging, Audit, Spondyloarthropathies

## Abstract

**Objectives:**

The assessment of axial spondyloarthritis international society (ASAS) has published recommendations on information to include when requesting and reporting MRIs for suspected or known axSpA. In a service improvement evaluation, we aimed to audit our current practice against the newly published recommendations.

**Methods:**

MRIs of the spine and sacroiliac joints performed under the inflammatory back pain protocol at The Leeds Teaching Hospitals NHS Trust between September and December 2023 were identified. Information included in the MRI request and radiology reports was extracted from the electronic health records.

**Results:**

Overall, 158 MRIs were evaluated. Of the MRI requests, age was included in 11.8% (*n* = 18), sex in 3.8% (*n* = 6); HLA-B27 in 22.2% (*n* = 35), back pain symptom duration in 18.9% (*n* = 30) and location in 45.6% (*n* = 72) and presence of inflammatory features in 62.7% (*n* = 99). For MRI report data: osteitis/bone marrow oedema was mentioned in 81.0% (*n* = 128), erosions in 58.8% (*n* = 93) and fat metaplasia in 6.3% (*n* = 10); active inflammation was mentioned in 77.8% (*n* = 123) and structural changes in 51.2% reports (*n* = 82). Over half (62.0%; *n* = 98) included a statement as to “whether the MRI was overall indicative of spondyloarthritis or not”.

**Conclusion:**

Despite the retrospective nature of this audit performed against newly published guidance, these results provide awareness, identifying key areas in need of improvement at our centre. Further, this exercise has strengthened the collaboration between rheumatologists and radiology colleagues, illustrating the value of the recent ASAS recommendations as a benchmark to guide further training and education for rheumatologists and radiologists, and improve clinical care provision in axSpA.

Key messages

- Data of clinical relevance are often missing on requests of MRI of the SIJ in axial spondyloarthritis.
- Over half of MRI reports at our centre include a statement as to whether findings are in keeping with axSpA.
- Although most MRI reports contained information on active inflammatory lesions, they often lacked detail on structural lesions.

## Introduction

Imaging is essential in the diagnosis and management of axial spondyloarthritis (axSpA), with magnetic resonance imaging (MRI) considered the method of choice to identify inflammatory and structural lesions [1, 2] in the spine and sacroiliac joints (SIJs), the hallmark anatomical areas of disease in axSpA. Indeed, positive imaging findings, either MRI or conventional radiography, increase clinician’s confidence in making the diagnosis of axSpA, a disease which relies heavily on clinician’s expertise. However, sensitivity and specificity of MRI are limited as some lesions may appear in healthy or non-inflammatory processes, including degenerative disease, exercise-related injuries or post-pregnancy physiological bone changes [3, 4]. Furthermore, in real-world practice, imaging interpretation relies on qualitative assessment by radiologists and rheumatologists. Historically, MRI acquisition differed between centres and geographical regions, resulting in variations in care for patients. However, in recent years, national and international guidelines on MRI acquisition in axSpA have been developed, resulting in improved acquisition standardization [1, 2].

Effective communication between rheumatologists and radiologists is essential to maximize the utility of imaging in axSpA [5]. A recent study demonstrated increased precision and specificity by radiologists when interpreting imaging of the SIJs if adequate clinical information was provided in the imaging request [6]. However, others have shown poor quality of imaging requests [7, 8]. Conversely, most radiologists work in non-specialized rheumatology centres or undergo limited training in musculoskeletal imaging, contributing insufficient awareness of the information required in an MRI report to inform treatment decision making. To improve the overall communication between rheumatologists and radiologists and standardize the information required from both specialties, the Assessment of SpondyloArthritis International Society (ASAS) have recently developed guidance on requesting [9] and reporting [10] MRI scans in suspected or known axSpA. Herein, we report the results of an audit performed at our centre aimed at assessing our current practice against the newly published guidance.

## Methods

A retrospective audit was undertaken at The Leeds Teaching Hospitals NHS Trust (LTHT), a large university hospital, which hosts the Leeds Specialist spondyloarthritis Service, a tertiary referral centre. After registration with the trust audit governance team (LOC1594), all MRIs of the spine and SIJs joints performed under the inflammatory back pain protocol, following national guidance [1] to diagnose suspected axSpA or monitor disease activity [11] in known axSpA between September and December 2023, were identified. Clinical information included in the clinicians’ requests and radiologists’ reports was retrieved from the electronic health record and compared against the aforementioned ASAS guidance [9, 10]. Musculoskeletal imaging (MSK), including rheumatology requests at LTHT, is reported by a dedicated team of specialist MSK radiologists; however, a small number were outsourced to a teleradiology company during the period covered by the audit, due to strain in health resources caused by the COVID-19 pandemic in previous years.

## Results

A total of 158 MRIs of the spine and SIJs were performed in 158 patients during the audit period. Most MRI scans (*n* = 133; 84.2%) were reported by the internal MSK radiologist team, with a small number (*n* = 25; 15.8%) being outsourced to an external reporting company. In terms of demographic and clinical data included on the MRI requests; age was included in 11.8% (*n* = 18), sex in 3.8% (*n* = 6); HLA-B27 in 22.2% (*n* = 35), presence of back pain in 87.3% (*n* = 138), symptom duration 18.9% (*n* = 30), pain location in 45.6% (*n* = 72), presence of inflammatory features (i.e. early morning stiffness, etc) in 62.7% (*n* = 99), suspected diagnosis in 94.3% (*n* = 149) and an alternative diagnosis in 17.1% (*n* = 27) requests (Table 1). For MRI report data (Table 2), osteitis/bone marrow oedema was mentioned in 81.0% of reports (*n* = 128), erosions in 58.8% of reports (*n* = 93) and fat metaplasia in 6.3% of reports (*n* = 10) (Table 2). The presence or absence of active inflammation was mentioned in 77.8% of reports (*n* = 123), structural changes in 51.2% reports (*n* = 82) and 62.0% of reports (*n* = 98) included a statement as to ‘whether the MRI was overall indicative of spondyloarthritis or not’. No differences were seen between scans reported by the internal team or those outsourced to an external company.

**Table 1:** Summary of data included in MRI requests of the spine and SIJs for suspected or known axSpA.

MRI Request results *n* = 158	
**Demographics**	
Age (*n* [%])	18 (11.4%)
Sex (*n* [%])	6 (3.8%)
HLA-B27 status (*n* [%])	35 (22.2%)
**History**	
Back pain present (*n* [%])	138 (87.3%)
Symptom duration (*n* [%])	30 (18.9%)
Location of pain (*n* [%])	72 (45.6%)
Inflammatory features (e.g. morning stiffness, pain improving with exercise) (*n* [%])	99 (62.7%)
**Alternative cause for findings**	
Physically demanding activity (*n* [%])	6 (3.8%)
Childbirth (*n* [%])	5 (3.2%)
**Clinical diagnosis**	
Suspected diagnosis of axSpA mentioned (*n* [%])	149 (94.3%)
Alternative diagnosis mentioned (e.g. lumbar stenosis, hypermobility spectrum disorder) (*n* [%])	27 (17.1%)

**Table 2:** Summary of data regarding identified lesions and report conclusions included in MRI reports for suspected or known axSpA.

MRI report results *n* = 158	
**Osteitis/bone marrow oedema**	
Presence (*n* [%])	128 (81.0%)
Location (*n* [%])	65 (97.0%)
Semi-quantitative (*n* [%])	60 (89.6%)
**Erosions**	
Presence (*n* [%])	93 (58.8%)
Location (*n* [%])	37 (88.0%)
Semi-quantitative (*n* [%])	29 (69.1%)
**Fat metaplasia**	
Presence (*n* [%])	10 (6.3%)
Location (*n* [%])	7 (100%)
Semi-quantitative (*n* [%])	4 (57.1%)
**Report conclusion**	
Non-axSpA findings mentioned (*n* [%])	102 (64.6%)
Active inflammatory changes (*n* [%])	123 (77.8%)
Structural changes (*n* [%])	82 (51.2%)
“Findings in keeping with axSpA?” (*n* [%])	98 (62.0%)
Alternative diagnosis (*n* [%])	117 (74.1%)
Other imaging recommended (*n* [%])	1 (0.6%)

## Discussion

The ASAS group has recently published guidance to standardize the quality of MRI requests and reports in suspected of known axSpA in clinical practice [9, 10]. This audit reports real-world data showing that overall information included on MRI requests and reports in axSpA at our centre was poor compared with the ASAS guidance. This was particularly the case when appraising the MRI requests written by rheumatologists, which were frequently missing key information, chiefly, reference to back pain history or suspected diagnosis. Similarly, albeit less frequently, data on structural MRI lesions and the radiologists’ overall interpretation of the findings were often missing from MRI reports.

Limitations of our report include the fact that it is a single centre and was conducted over a short time frame. Furthermore, it is retrospectively addressing recently published guidance which has had only limited dissemination to date. In this light, our results are similar to those published in a recent, larger report by Hadsbjerg *et al.* [12], which compared 913 MRI reports from centres within 5 European countries, identifying a large gap between clinical reporting of SIJ MRIs and the recently published recommendations [10]. Like our data, 73% of local MRI reports included information as to the presence or absence of inflammatory lesions and 39% as to the presence or absence of structural lesions. Yet, in the context of any findings, only 24% included a statement as to ‘whether the MRI was overall indicative of spondyloarthritis or not’, arguably the most relevant information to be obtained from the MRI report for the requesting clinicians, which is much lower than that reported from our centre (62%). This discrepancy may be explained by the fact that our service is located within a large tertiary rheumatology centre, which includes a dedicated MSK radiology team, whereas the Hadsbjerg’s study included a variety of rheumatology centres from across Europe. Additionally, the authors report over a larger time frame (2005 to 2022), when clinical practice may have changed over time, whereas our audit assesses MRIs requested over a more recent (2023) and shorter (4-month) period. Of note, a 2024 freedom of information report in the UK showed significant improvement in radiologist awareness of the term axSpA, and imaging findings suggestive of axSpA compared with a previous survey, performed in 2017 [13, 14]. This was predominantly attributed to the publication of the 2019 British Society for Spondyloarthritis (BRITSpA) MRI acquisition guidelines [1], which has been regularly disseminated between rheumatologists and radiologists in the UK. Our findings further highlight the benefits of developing national specialist groups for the advancement of spondyloarthritis care.

The main strength of this work is that it was performed as a cross-specialty initiative, fostering collaboration and improving the dialogue between the MSK radiology and rheumatology teams in our centre. Further, it has provided significant insight, highlighting areas of educational need for both teams. Overall, the MRI request data in our centre was poor, particularly with regard to clinical history, which is key and may impact upon imaging interpretation. Whilst data related specifically to rheumatology imaging or MRI requests are overall sparse, our findings are in keeping with the available literature [7, 8], which highlights poor imaging requests throughout multiple departments, imaging modalities and clinical indications. Next steps after enhanced internal training at our centre, will include a repeat audit to assess progress and improvement of our processes.

The demand for imaging has increased significantly in recent years globally [15]. To maximize the benefit to patients, adequate clinical information is required to ensure that the right modality, anatomical coverage and sequences are performed to answer the underlying clinical question and allow radiologists to accurately interpret findings. However, concerns have been raised as to whether providing detailed clinical information in radiology requests may introduce confirmation bias when radiologists report imaging studies, thus influencing the interpretation of the findings, leading to under- or overdiagnosis [16]. In this sense, educational tools are welcome to help improve and standardize clinical practice. The Reason for Exam Imaging Reporting and Data System (RI-RADS) tool [17] ranks image requests based on the quality and extent of information provided. A single-centre study from the Netherlands [7] highlighted >50% of all imaging requests were inadequate; similarly, a study in Italy [8] found 92% of all inpatient imaging requests were of inadequate quality. In these studies, pre-operative imaging requests were associated with poorer quality, whereas imaging for infection/inflammation was associated with better request quality. Multiple factors are likely to contribute to poor imaging, including a lack of training, a high burden of overall administrative tasks and clinician workload. These data highlight that significant work is required across all healthcare professionals to improve the quality of imaging requests and maximize the utility of imaging to improve patient care.

Compounding the challenges of increased demand for imaging, there has been a shortfall in the number of radiologists trained to match this increased demand [18]. Therefore, the development of tools to streamline reporting and minimize risk of clinician misinterpretation of findings is warranted. The Reporting and Data System (RADS) utilized in radiology serve as a standardized reporting system that organizes expert decision-making processes into a clear and structured flow [19] and helps translate complex findings into understandable terms by providing probabilities for specific diagnoses, facilitating and standardizing radiology reports. RADS is most commonly used in the context of suspected malignancy, for example, multiparametric MRI of the prostate is scored on a scale between 1 and 5, with 1 representing a very low likelihood of malignancy and 5 a very high likelihood of malignancy, which then guides the next steps in patient management. Additionally, advances in artificial intelligence may further optimize workflows and improve imaging interpretation [20]. Implementing these systems for imaging in suspected axSpA may help to improve reporting standards and translatability of imaging findings to patient management.

In conclusion, this audit has highlighted key areas in need of improvement at our service when considering imaging in axSpA. In this sense, the recent ASAS recommendations for the requests and reports of MR imaging for patients with suspected or known axSpA provide a benchmark to guide further training and education for both rheumatologists and radiologists. Further, disseminating and implementing these recommendations can enhance an improved dialogue and communication between rheumatology and radiology teams, ultimately improving patient care in axSpA.

## Data Availability

The data underlying this article cannot be shared publicly due as this is routinely collected clinical data.
